# Lysine at position 329 within a C-terminal dilysine motif is crucial for the ER localization of human SLC35B4

**DOI:** 10.1371/journal.pone.0207521

**Published:** 2018-11-20

**Authors:** Bożena Bazan, Maciej Wiktor, Dorota Maszczak-Seneczko, Teresa Olczak, Beata Kaczmarek, Mariusz Olczak

**Affiliations:** Faculty of Biotechnology, University of Wrocław, Wrocław, Poland; Duke University School of Medicine, UNITED STATES

## Abstract

SLC35B4 belongs to the solute carrier 35 (SLC35) family whose best-characterized members display a nucleotide sugar transporting activity. Using an experimental model of HepG2 cells and indirect immunofluorescent staining, we verified that SLC35B4 was localized to the endoplasmic reticulum (ER). We demonstrated that dilysine motif, especially lysine at position 329, is crucial for the ER localization of this protein in human cells and therefore one should use protein C-tagging with caution. To verify the importance of the protein in glycoconjugates synthesis, we generated SLC35B4-deficient HepG2 cell line using CRISPR-Cas9 approach. Our data showed that knock-out of the *SLC35B4* gene does not affect major UDP-Xyl- and UDP-GlcNAc-dependent glycosylation pathways.

## Introduction

Glycosylation belongs to one of the most frequent posttranslational modifications of macromolecules. Synthesis and modification of glycan moiety is performed by glycosyltransferases located in the lumen of the endoplasmic reticulum (ER) and Golgi apparatus. Glycosyltransferases use sugars activated by the addition of a nucleoside mono- or diphosphate (UDP, GDP, or CMP) as substrates. Nucleotide sugars are synthesized in the cytosol [1], and in the case of CMP-sialic acid (CMP-Sia) in the nucleus [2]. To be available for glycosyltransferases, they must be transported into the ER or Golgi apparatus. This function is performed by nucleotide sugar transporters (NSTs), which are integral membrane proteins [3,4]. Most bioinformatics algorithms predict that NSTs possess even number of transmembrane domains, which results in the N- and C- termini being exposed to the cytosol. However, membrane topology has been experimentally determined for the murine CMP-Sia transporter only [5]. Several studies have demonstrated that NSTs function in the form of homodimers [5–8] or higher homooligomers [9]. Moreover, there are several reports demonstrating complex formation between various NSTs as well as between NSTs and functionally related glycosyltransferases [10–13].

The first characterized NSTs were demonstrated to be specific for the translocation of a single nucleotide sugar [6,7,14], but later, multi-substrate NSTs were described [15,16]. Among them is SLC35 member B4 protein (SLC35B4). In humans, the *SLC35B4* gene is mapped to chromosome 7q33 and encodes two splice variants: a longer version (encoding a protein of 331 amino acids) [17,18] and a shorter version (encoding a protein of 231 amino acids) [18]. According to Ashikov *et al*. [17], vesicles derived from *Saccharomyces cerevisiae* cells expressing the longer splice variant of the human SLC35B4 protein showed specific uptake of UDP-*N*-acetylglucosamine (UDP-GlcNAc) and UDP-xylose (UDP-Xyl). Roy *et al*. [19] demonstrated that the *S*. *cerevisiae* SLC35B4 homolog (YEA4) transported UDP-GlcNAc. Microsomes derived from V79 cells (Chinese hamster lung fibroblasts) overexpressing both splice variants of the SLC35B4 transporter showed specific uptake of UDP-glucuronic acid (UDP-GlcA), but only after preloading of microsomes with UDP-GlcNAc [19]. Ishikawa *et al*. [20] found that *Drosophila melanogaster* SLC35B4/YEA4 homolog (Efr) also transported GDP-fucose (GDP-Fuc).

## Materials and methods

### Cell cultures and stable transfection

HepG2 cells (cell collection of Institute of Immunology and Experimental Therapy, Polish Academy of Sciences, Wrocław, Poland) were maintained under humidified atmosphere (37°C and 5% CO_2_) in Minimum Essential Medium Eagle (MEM, Biowest). Medium was supplemented with 10% fetal bovine serum (FBS), 100 U/ml penicillin, 100 μg/ml streptomycin and 2 mM L-glutamine (Cytogen). Cells were stably transfected with the respective plasmids using FuGENE 6 transfection reagent (Promega) according to the manufacturer’s protocol. Stable transfectants overexpressing SLC35B4 tagged with HA at the C-terminus, N-terminus or modified variants of the recombinant N-tagged protein were selected in complete media containing 200 μg/ml zeocin (InvivoGen). Cells were analyzed for overexpression of HA-tagged proteins using indirect immunofluorescent staining.

### Cloning and site-directed mutagenesis

Total RNA was isolated from HeLa cells, and cDNA encoding human SLC35B4 protein (NCBI accession number AAH08413) was synthesized as described previously [21]. PCR products were amplified using primers listed in Table 1, purified, digested with BamHI and NheI restriction enzymes and cloned into the pSelect-zeo vector (InvivoGen). All plasmids constructed and used in this study are listed in Table 2. Amino acid substitutions K329A, K330A or K329A/K330A were introduced to the *SLC35B4* gene using QuikChange Multi Site-Directed Mutagenesis Kit (Agilent Technologies) and primers listed in Table 1. All constructs were subsequently verified by DNA sequencing (Genomed, Warszawa, Poland).

**Table 1:** List of primers designed and used in this study.

Name	Oligonucleotide sequence 5’→3’	Description
HA_HsSLC35B4_Bam_F	AGC*GGATCC*CCAATGGCTTACCCATACGACGTACCAGACTACGCAATGCGCCCGGCCTTGG	Primers used to amplify insert to overexpress recombinant HA-SLC35B4
HsSLC35B4_Nhe_STOP_R	CAG*GCTAGC*TCAGTTCTTCTTGCTGTCCTTCTG	
HsSLC35B4_Bam_F	AGC*GGATCC*CCAATGCGCCCGGCCTTGG	Primers used to amplify insert to overexpress recombinant SLC35B4-HA
HA_HsSLC35B4_Nhe_R	CAG*GCTAGC*TCATGCGTAGTCTGGTACGTCGTATGGGTAGTTCTTCTTGCTGTCCTTCTGAGG	
hsSLC35B4_K329A	GAGCCTCAGAAGGACAGC**GC**GAAGAACTGACTAGCTGG	Primers used for site-directed mutagenesis
hsSLC35B4_K330A	CTCAGAAGGACAGCAAG**GC**GAACTGACTAGCTGGCC	
hsSLC35B4_K329AK330A	TGAGCCTCAGAAGGACAGC**GC**G**GC**GAACTGACTAGCTGGC CAG	
hsSLC35B4_mRNA_F	ATGCGCCCGGCCTTGGCG	Primers used to amplify region encoding fragment of SLC35B4 in mRNA
hsSLC35B4_mRNA_R	CCATCCTTGCTGACATCAG	
hsSLC35B4_DNA_F	GTCCTCTCTGGCGGAGCTGCCTGG	Primers used to amplify region encoding fragment of SLC35B4 in genomic DNA
hsSLC35B4_DNA_R	TGGTGAAGGAACCTTGGTTCTG	

DNA sequence encoding HA tag is underlined. Sites recognized and cleaved by restriction enzymes are shown in italic. Nucleotide substitutions are shown in bold.

**Table 2:** List of expression plasmids constructed and used in this study.

Plasmid name	Original vector	Insert
pSelect-HA-SLC35B4	pSelect-zeo (InvivoGen)	Human SLC35B4 with HA tag at the N-terminus
pSelect-SLC35B4-HA	pSelect-zeo (InvivoGen)	Human SLC35B4 with HA tag at the C-terminus
pSelect-HA-SLC35B4-K329A	pSelect-HA-SLC35B4	Human SLC35B4 K329A with HA tag at the N-terminus
pSelect-HA-SLC35B4-K330A	pSelect-HA-SLC35B4	Human SLC35B4 K330A with HA tag at the N-terminus
pSelect-HA-SLC35B4-K329AK330A	pSelect-HA-SLC35B4	Human SLC35B4 K329AK330A with HA tag at the N-terminus

### Construction and analysis of SLC35B4 knock-out cell line

HepG2 cells were transfected with a mixture of human SLC35B4 double nickase plasmids according to the manufacturer's instructions (Santa Cruz Biotechnology) and selection was performed in MEM complete medium supplemented with 1 μg/ml puromycin (InvivoGen). Twelve independent clones overexpressing GFP (the second selection marker in the utilized CRISPR-Cas9 system) were isolated. To verify gene knock-out, total RNA and genomic DNA were isolated from cells and RT-PCR and PCR reactions were performed, respectively, using HepG2 wild-type cells as a control. To identify mutations introduced by the CRISPR-Cas9 approach, PCR products obtained using cDNA as a template were cloned into the pJET1.2/blunt vector according to the manufacturer's instructions (Thermo Fisher Scientific) and sequenced using pJET1.2 forward and pJET1.2 reverse sequencing primers (Thermo Fisher Scientific). PCR products obtained from genomic DNA as a template were sequenced directly without cloning using hsSLC35B4_DNA_F and hsSLC35B4_DNA_R primers (Table 1).

### Immunofluorescent imaging

Cells were seeded onto glass 8-well microscope slides (Merck) coated with 0.01% poly-L-lysine (Sigma-Aldrich), fixed and subsequently treated as described in details previously [22]. Briefly, cells were washed 3 more times with PBS. Non-specific binding sites were blocked with blocking solution containing 1% (w/v) BSA and 0.1% (w/v) saponin in PBS for 1 hour at RT. Subsequently, slides were incubated at 37°C for 1 h with primary antibodies (Table 3) in blocking solution, washed with blocking buffer and incubated at 37°C for 1 h with goat anti-rabbit or anti-mouse IgG Alexa Fluor 488-, Alexa Fluor 568 or Alexa Fluor 633-conjugated secondary antibodies (Molecular Probes) diluted 1:200 in blocking solution. Cell nuclei were counterstained with 4',6-diamidino-2-phenylindole (DAPI, Sigma-Aldrich). Slides were mounted onto glass coverslips using fluorescence mounting medium (Dako), and analyzed using a ZEISS LSM 510 confocal microscope and ImageJ software 1.48v (NIH).

**Table 3:** List of primary antibodies used for immunofluorescent analysis.

Antibody	Clonality	Dilution	Host	Company
anti-HA	Polyclonal	1:500	Rabbit	Abcam
anti-HA	Monoclonal	1:500	Mouse	Thermo Fisher Scientific
anti-calnexin	Polyclonal	1:100	Rabbit	Abcam
anti-syntaxin 16	Monoclonal	1:200	Rabbit	Abcam
anti-GM130	Monoclonal	1:200	Mouse	BD Biosciences
Anti-LAMP1	Polyclonal	1:100	Rabbit	Abcam
Anti-TNG38	Monoclonal	1:25	Mouse	Santa Cruz
Anti-ERGIC	Monoclonal	1:25	Mouse	Santa Cruz

### Analysis of glycoconjugates

To analyze *N*-glycans, permethylation of isolated oligosaccharides without desialylation was carried out [23,24] and derivatives analyzed using mass spectrometry as described below. To characterize *O*-glycans, modified procedure by Kudelka *et al*. [24] was used. Briefly, wild-type and SLC35B4-deficient HepG2 cells (8×10^5^) were seeded onto 10-cm dishes (Falcon) in 10 ml of a complete growth medium containing 10% FBS. After 24 hours medium was replaced with 10 ml of medium with reduced (5%) FBS content, supplemented with Ac_3_GalNAcBn (50 μM) and cells were grown for additional 72 hours. Next, conditioned media were collected, centrifuged (1000×*g*) in order to remove cells detached upon medium aspiration and the resulting supernatants were subjected to glycan extraction procedure [24] with the exception that instead Sep-Pak C18 column (Waters), 3-ml Supelclean LC18 column (Supelco) was used.

For immunodetection of selected proteoglycans, cell lysates were subjected to SDS-PAGE using 8% polyacrylamide gels and after transferring onto nitrocellulose membranes visualized with mouse anti-chondroitin-4-sulfate (1:5000, Millipore) and mouse anti-keratan sulfate (1:5000, Millipore) antibodies followed by anti-mouse IgG antibody conjugated with HRP (1:10000, Promega). For detection of chondroitin-4-sulfate, before SDS-PAGE cell lysates were treated with 0.2 U/ml chondroitinase ABC (Sigma Aldrich) in 50 mM Tris-HCl, containing 60 mM sodium acetate for 2 h at 37°C. Protein loading was assessed using Coomassie Brilliant Blue G-250 (CBB) staining.

### Mass spectrometry

Positive ion MALDI mass spectra were recorded on the Voyager-Elite (PerSeptive Biosystems) instrument equipped with nitrogen laser (337 nm) in reflector mode at an acceleration voltage of 20 kV (Center for Molecular and Macromolecular Research, Polish Academy of Sciences, Łódź, Poland). Vacuum-dried glycan samples were dissolved in 10–20 μl of either H_2_O or 50% MeOH depending on their solubility. Glycan solution (5 μl) was combined with matrix (5 μl) and the resulting mixture (1 μl) was spotted on the MALDI plate and allowed to air dry. As a matrix, 20 mg/ml 2,5-dihydroxybenzoic acid (Sigma-Aldrich) dissolved in 20 mM sodium acetate buffer, 20% MeCN was used. Mass spectrum was accumulated from at least 100 laser shots and processed by Data Explorer ver. 4 (Applied Biosystems) and Microsoft Office 2013.

## Results and discussion

**Fig 1:**
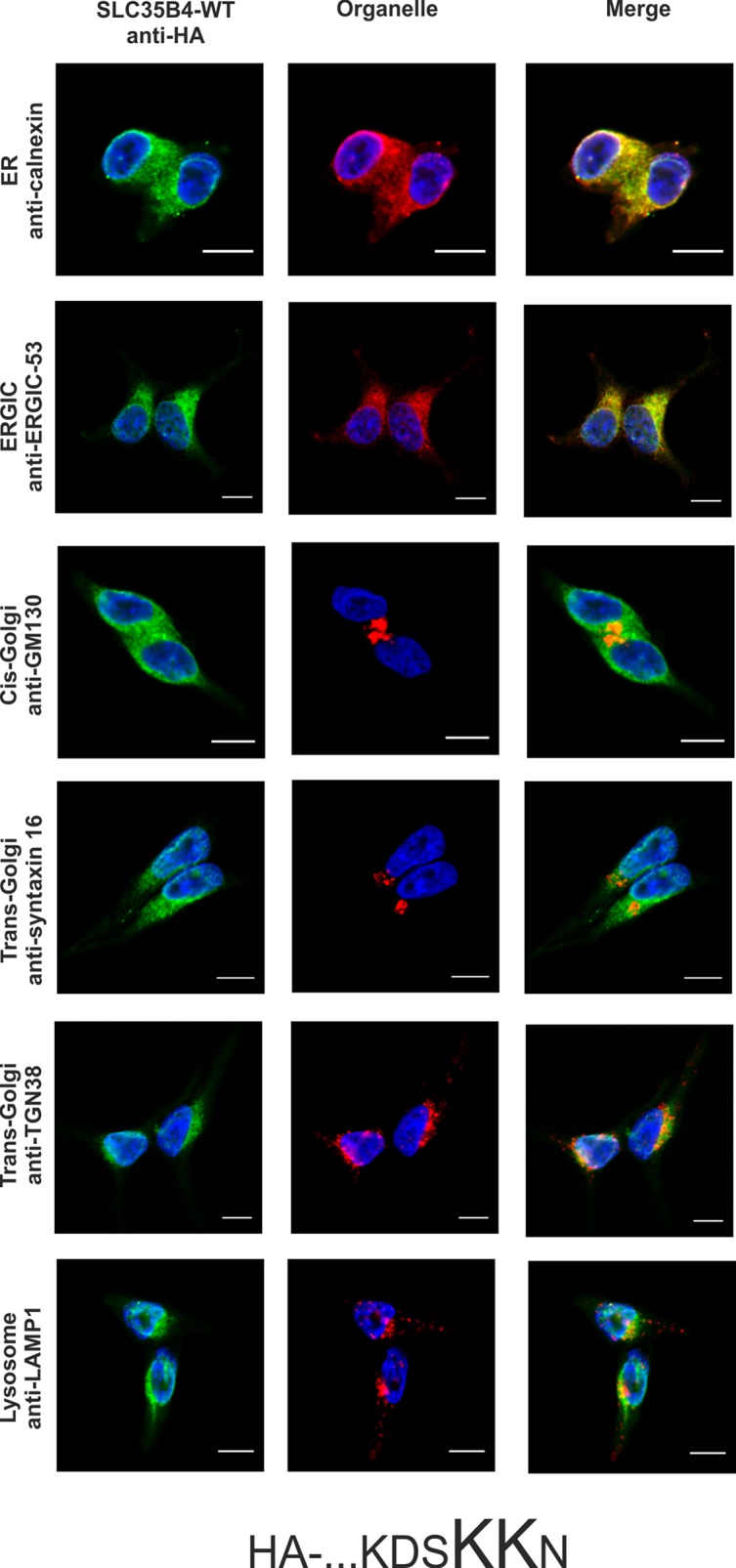
Subcellular localization of HA- …KDSKKN, human SLC35B4 with HA-epitope at the N-terminus in the wild-type HepG2 cells. Stably transfected wild-type (SLC35B4-WT) cells, overexpressing SLC35B4 variants, were subjected to indirect immunofluorescent staining with anti-HA (green) and anti-organelle markers (Table 3) (red) antibodies. Cell nuclei were counterstained with DAPI. Scale bar 10 μm.

**Fig 2:**
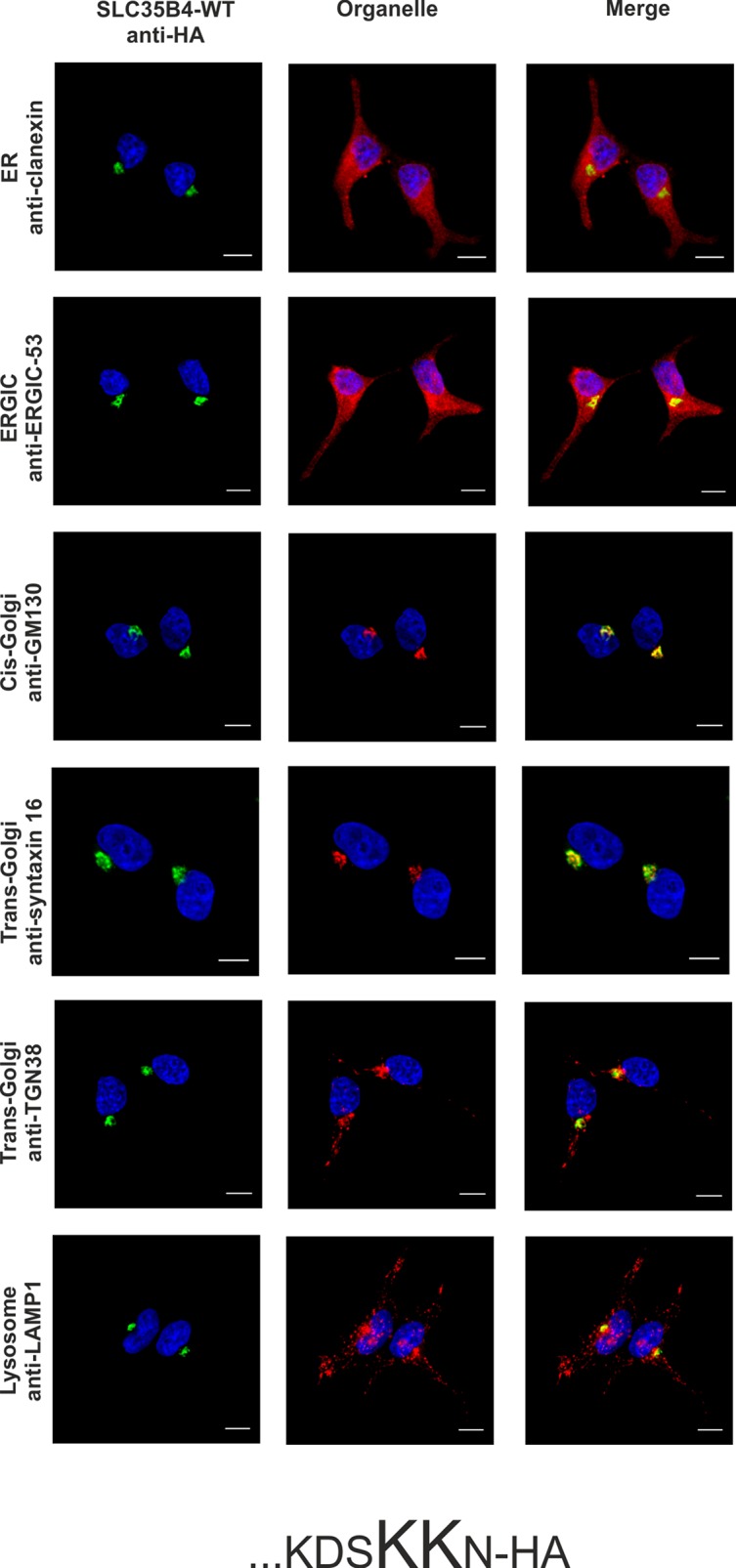
Subcellular localization of …KDSKKN-HA, human SLC35B4 with HA-epitope at the C-terminus in the wild-type HepG2 cells. Stably transfected wild-type (SLC35B4-WT) cells, overexpressing SLC35B4 variants, were subjected to indirect immunofluorescent staining with anti-HA (green) and anti-organelle markers (Table 3) (red) antibodies. Cell nuclei were counterstained with DAPI. Scale bar 10 μm.

Literature data has shown that transiently overexpressed human SLC35B4 localized in CHO-K1 cells to the Golgi apparatus [17]. In contrast, homologous proteins from *S*. *cerevisiae* (YEA4) and *D*. *melanogaster* (Efr) have been found in the ER of yeasts and fly epithelium of third instar wing discs, respectively [19,20]. Our preliminary studies demonstrated that SLC35B4 co-localized exclusively with the ER marker when overexpressed in Madin-Darby canine kidney (MDCK) wild-type and MDCK mutant cells resistant to *Ricinus communis* agglutinin (MDCK-RCA^r^) [21]. These contradictory results prompted us to further clarify the subcellular localization of the human SLC35B4 longer splice variant using model human HepG2 cells (both the wild-type cells and knock-out cells lacking functional *SLC35B4* gene) stably overexpressing HA-tagged protein and indirect immunofluorescent imaging. We have chosen this cell line because our preliminary studies showed that mRNA encoding SLC35B4 is expressed at different levels in cell lines and human tissues, with higher levels in liver tissue among others ([25] and our unpublished data). To detect marker proteins specific for the ER, Golgi apparatus, ER-Golgi-intermediate compartment (ERGIC), and lysosomes specific antibodies were used (Table 3). The SLC35B4 longer splice variant contains a C-terminal dilysine motif (KDSKKN), which is slightly different than the classic motifs KKXX or K*X*K*XX*, which are known to cause ER retention of some membrane proteins [26,27]. Here we found that subcellular localization of the recombinant SLC35B4 strongly depends on the position of the attached fusion tag. Similar to our previous report [21], N-terminal tagging resulted in the ER localization of the recombinant protein (Fig 1). Our finding is in contrast to data reported by Ashikov *et al*. [17], who demonstrated Golgi localization of N-terminally FLAG-tagged SLC35B4 in CHO-K1 cells. In our case, C-terminal tagging shifted the majority of the protein towards the Golgi apparatus (Fig 2). This suggests that the dilysine motif present within the C-terminus of SLC35B4 confers localization of the latter in the ER. This might also explain why Mkhikian *et al*. [28] observed Golgi localization in Jurkat T cells when SLC35B4 was DDK tagged at the C-terminus. Our findings are in agreement with those reported by Ishikawa *et al*. [20], who observed that deletion of the KKVE C-terminal region of *D*. *melanogaster* Efr resulted in mislocalization of the protein, which was predominantly found in the Golgi apparatus. Our data are in contrast to those reported for *S*. *cerevisiae* YEA4 protein, which although HA tagged at the C-terminus (possessing the IKKSK motif), localized to the ER [19]. In addition, some published data are difficult to assess because staining for ER markers is not shown [17,18,28].

**Fig 3:**
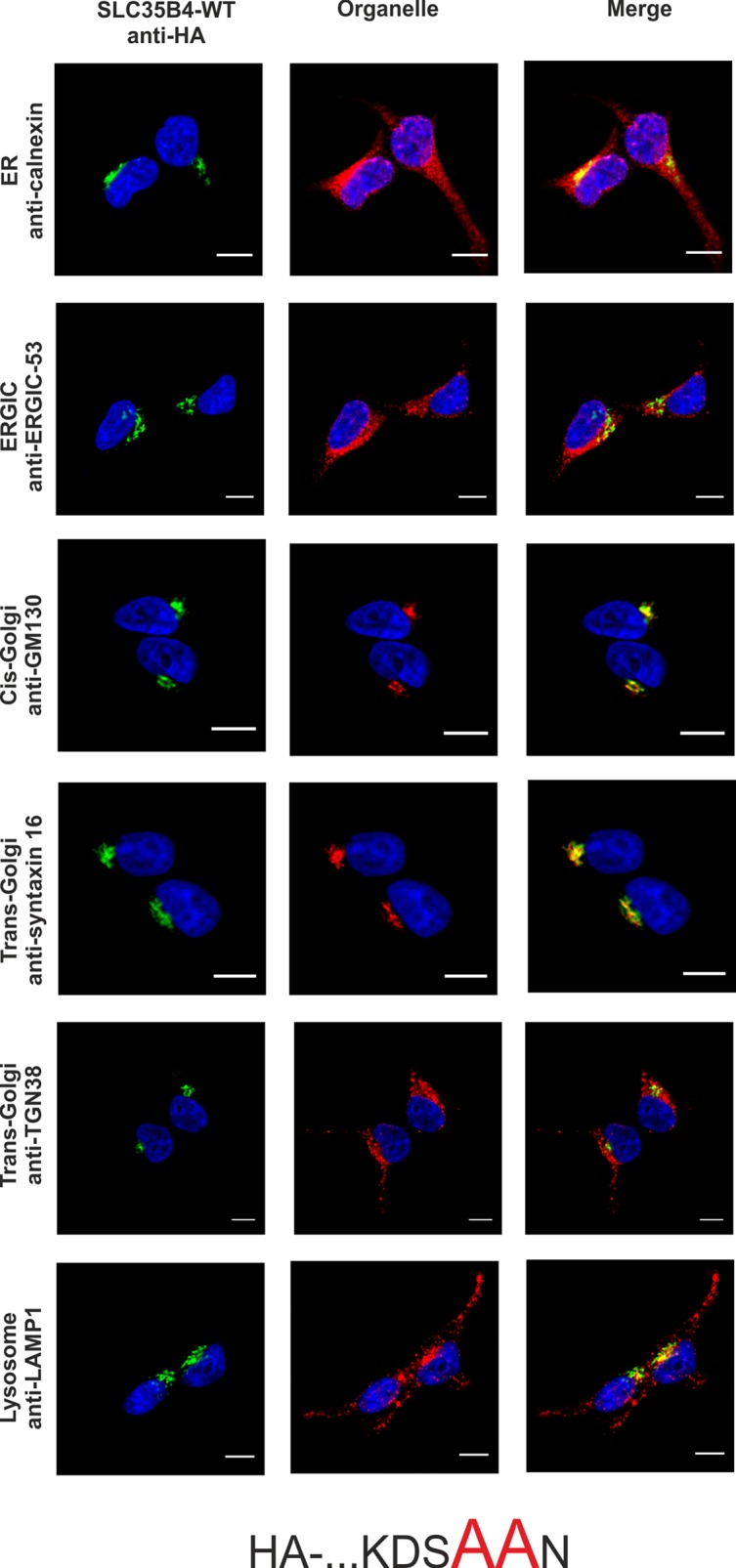
Subcellular localization of HA- …KDSAAN, human SLC35B4 K329AK330A with HA-epitope at the N-terminus in the wild-type HepG2 cells. Stably transfected wild-type (SLC35B4-WT) cells, overexpressing SLC35B4 variants, were subjected to indirect immunofluorescent staining with anti-HA (green) and anti-organelle markers (Table 3) (red) antibodies. Cell nuclei were counterstained with DAPI. Scale bar 10 μm.

**Fig 4:**
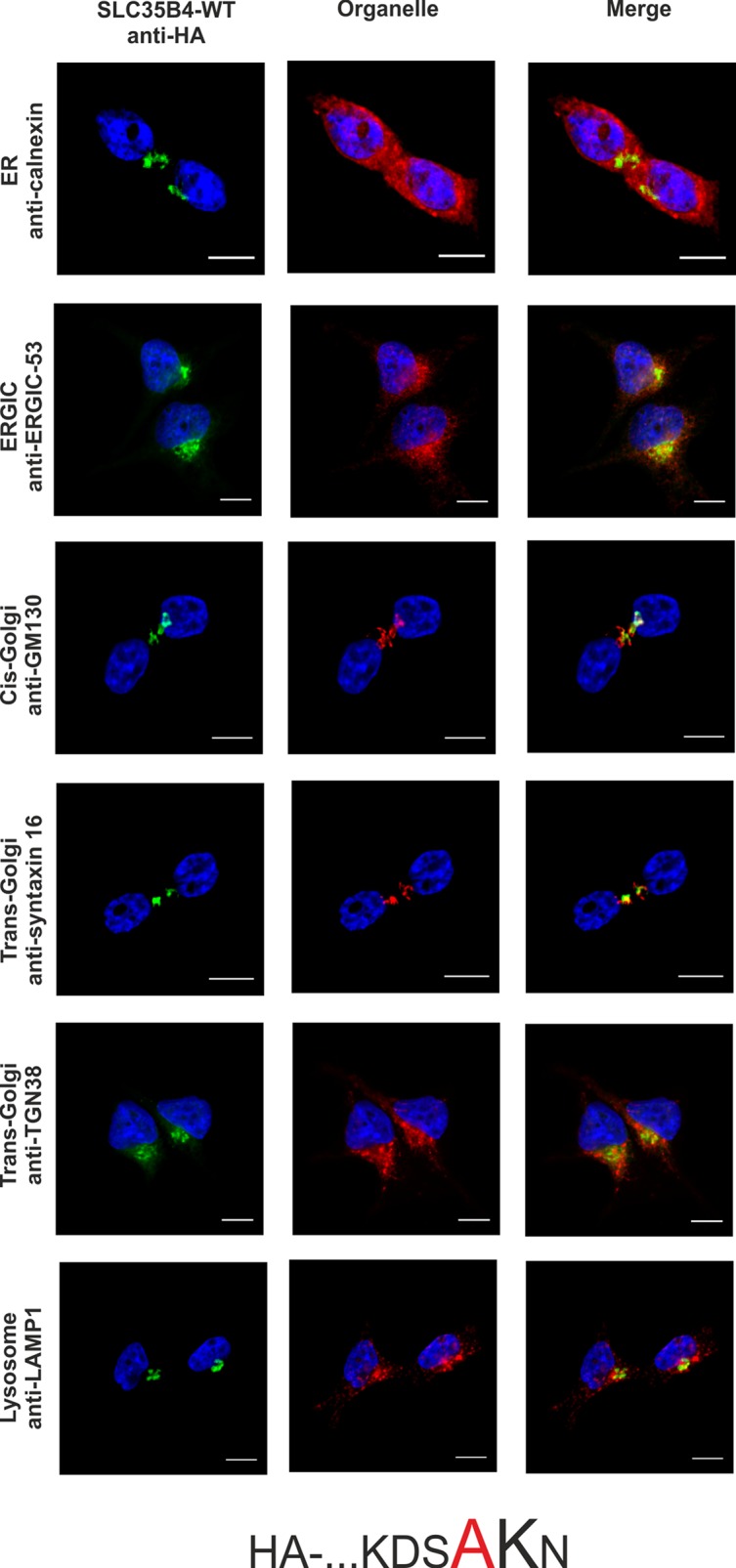
Subcellular localization of HA- …KDSAKN, human SLC35B4 K329A with HA-epitope at the N-terminus in the wild-type HepG2 cells. Stably transfected wild-type (SLC35B4-WT) cells, overexpressing SLC35B4 variants, were subjected to indirect immunofluorescent staining with anti-HA (green) and anti-organelle markers (Table 3) (red) antibodies. Cell nuclei were counterstained with DAPI. Scale bar 10 μm.

**Fig 5:**
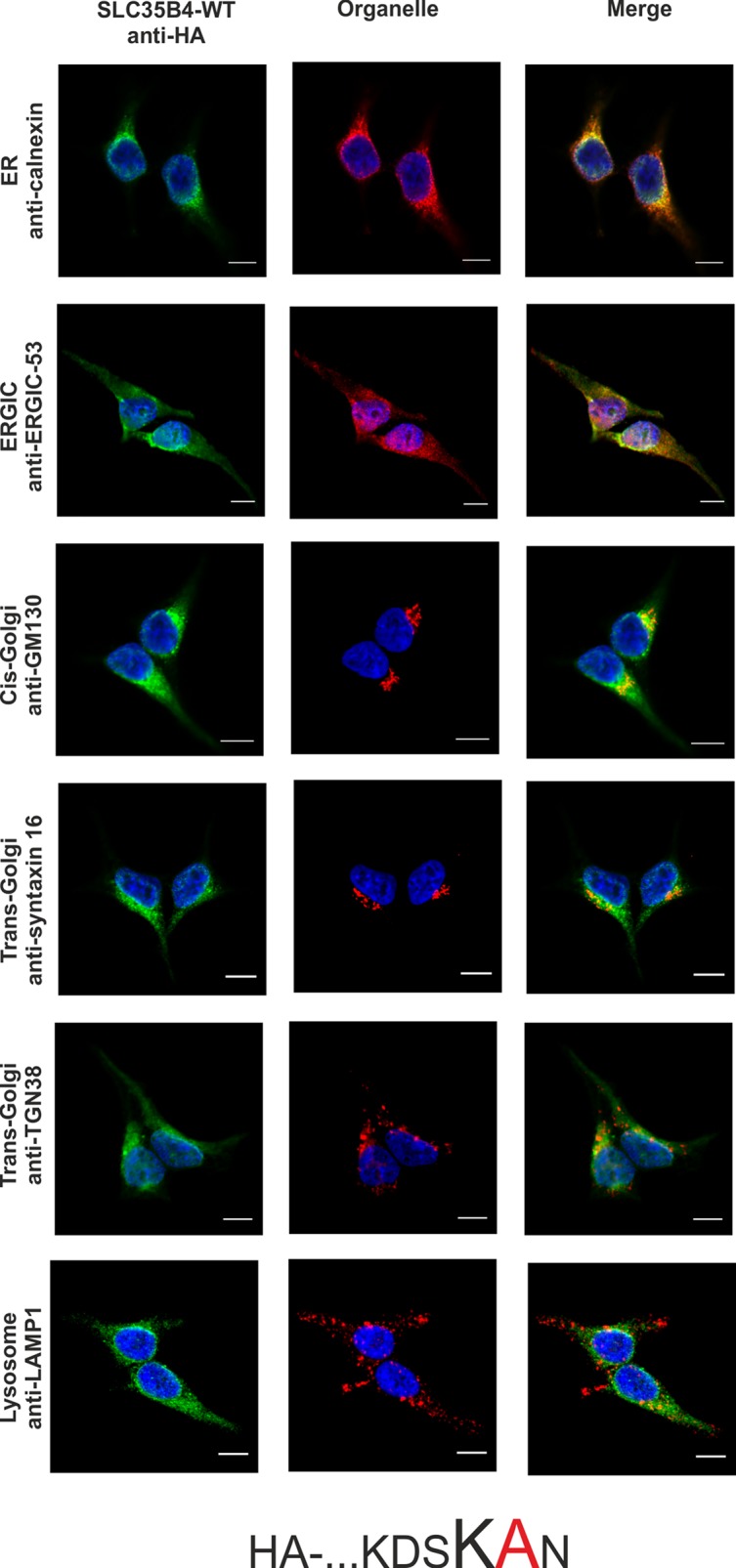
Subcellular localization of HA-…KDSKAN, human SLC35B4 K330A with HA-epitope at the N-terminus in the wild-type HepG2 cells. Stably transfected wild-type (SLC35B4-WT) cells, overexpressing SLC35B4 variants, were subjected to indirect immunofluorescent staining with anti-HA (green) and anti-organelle markers (Table 3) (red) antibodies. Cell nuclei were counterstained with DAPI. Scale bar 10 μm.

To confirm the importance of a dilysine motif for SLC35B4 ER localization we replaced individual or both lysine residues with alanine residues. When both lysine residues were substituted, the resulting protein was substantially re-localized, mainly to the Golgi apparatus (Fig 3). The pattern of the overexpressed protein was similar to that observed for the localization of the C-terminally tagged SLC35B4 (Fig 2). Importantly, we found that the first lysine (K329) within the dilysine motif is crucial for the ER localization of SLC35B4 in human cells (Fig 4), while the second lysine (K330) appears to be non-essential (Fig 5).

Taking into account the inconsistency between different reports on the subcellular localization of the human SLC35B4 and its homologs, the biological significance of this protein in mammalian cells should be reconsidered. Some previously published data showing that human SLC35B4 localized to the Golgi apparatus suggested its involvement in delivering UDP-GlcNAc and UDP-Xyl for proteoglycan synthesis [17]. Our [21] and others’ [19,20] results demonstrating ER localization of SLC35B4 do not support this hypothesis since the main synthesis steps of glycosaminoglycan moieties of proteoglycans, which require UDP-Xyl, occur mainly in the Golgi apparatus [29]. In the majority of published studies transport activity of NSTs was examined in a yeast heterologous system. Using this model SLC35B4 has been identified as a dual-specificity transporter [17]. Therefore one might suggest two hypotheses: (i) assuming that SLC35B4 indeed transports UDP-GlcNAc and UDP-Xyl, both substrates are utilized in less common metabolic pathway(s) in the ER; or (ii) the substrate specificity was mistakenly identified and therefore the yeast heterologous system is not an appropriate approach to explore function of putative glycosylation-related proteins of mammalian origins. The first scenario could be taken into consideration assuming that vesicular transport of nucleotide sugars between the ER and Golgi apparatus does not exist. If the second scenario is true, substrate specificity of a number of NSTs, and in particular those exhibiting multi-substrate specificity (reviewed in [4]), determined using yeast heterologous system should be questioned.

Several reports suggested that SLC35B4 could also transport other nucleotide sugars. Kobayashi *et al*. [18] observed that the protein transported UDP-GlcA only after preloading of microsomes derived from ovary cells with UDP-GlcNAc. However, this activity was the lowest among others reported. *D*. *melanogaster* Efr (a homolog of human SLC35B4) has been suggested to transport GDP-Fuc [20], thus contributing to the *O*-fucosylation of Notch [30]. Although Roy *et al*. [19] and Ishikawa *et al*. [20] observed ER localization of the yeast YEA4 (a homolog of human SLC35B4) and *Drosophila* Efr, respectively, both proteins are homologous proteins produced by different organisms and therefore they may differ in biological activity as compared to mammalian SLC35B4. It has been reported that the expression of the human *SLC35B4* gene is relatively high in the liver ([25,31] and our unpublished data) and its product might control hepatic insulin resistance and gluconeogenesis [31]. However, knock-down of the corresponding gene in HepG2 cells using siRNA showed increased gluconeogenesis, which was in contrast to *in vivo* and *in vitro* observations in mouse-based studies [32]. In light of all these findings, it is still necessary to find a native function of SLC35B4.

Although we clearly showed that when tagged at the N-terminus, the recombinant SLC35B4 localizes exclusively to the ER, we were unable to draw the same conclusion for the endogenous protein, since the specificity of commercial antibodies targeting human SLC35B4 we tested to date was highly dissatisfying. Therefore, a possibility that a subset of endogenous SLC35B4 resides in the Golgi membrane and supplies the respective enzymes with substrates for the attachment of Xyl and GlcNAc to macromolecules cannot be completely excluded. However, an ER-localized NST could also support Golgi-dependent glycosylation provided that vesicular transport of nucleotide sugars between these two organelles takes place. Such a passive movement of activated sugars was suggested by Kabuss *et al*. [33] to explain the phenotypic reversion of cells deficient in UDP-Gal import into the Golgi lumen by the ER-localized splice variant of UDP-Gal transporter. However, such phenomenon has never been proved. All these controversies encouraged us to undertake a more conclusive attempt involving targeted inactivation of the *SLC35B4* gene followed by comprehensive assessment of glycosylation capacity displayed by the resulting transfectants.

**Fig 6:**
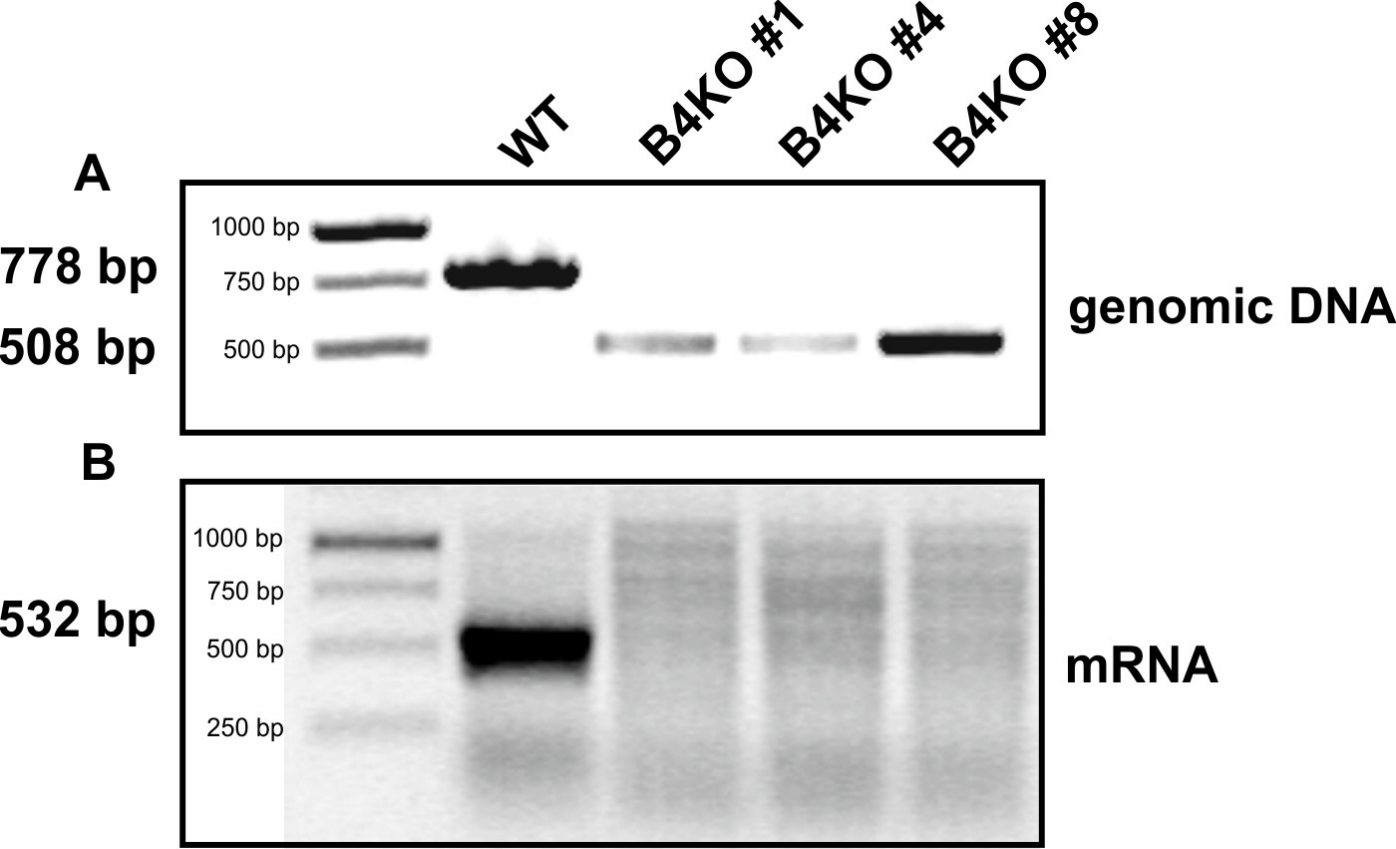
CRISPR-Cas9 knock-out of the *SLC35B4* gene in HepG2 cells. Genomic DNA (A) and total RNA (B) were isolated from SLC35B4-deficient (B4KO) and wild-type (WT) HepG2 cells and PCR (genomic DNA) or RT-PCR (mRNA) reaction was performed using *SLC35B4* gene-specific primers. Products were separated in 2% agarose gel, containing ethidium bromide.

To verify the suggested UDP-Xyl/UDP-GlcNAc transport activity of endogenous SLC35B4 in its native environment, knock-out of the corresponding gene was carried out in HepG2 cells using the CRISPR-Cas9 double nickase approach. Several independent clones were generated and knock-out of the *SLC35B4* gene was verified at both genomic DNA and mRNA levels. As shown in Fig 6 and S1 Fig, genomic DNA derived from the three examined knock-out clones possesses 270-bp deletion, encompassing region covering part of 5’-UTR region and a substantial part of exon 1, including ATG start codon. These results were confirmed by RT-PCR analysis of transcript encoding SLC35B4, demonstrating no specific amplification products only in the three knock-out clones examined (Fig 6).

**Fig 7:**
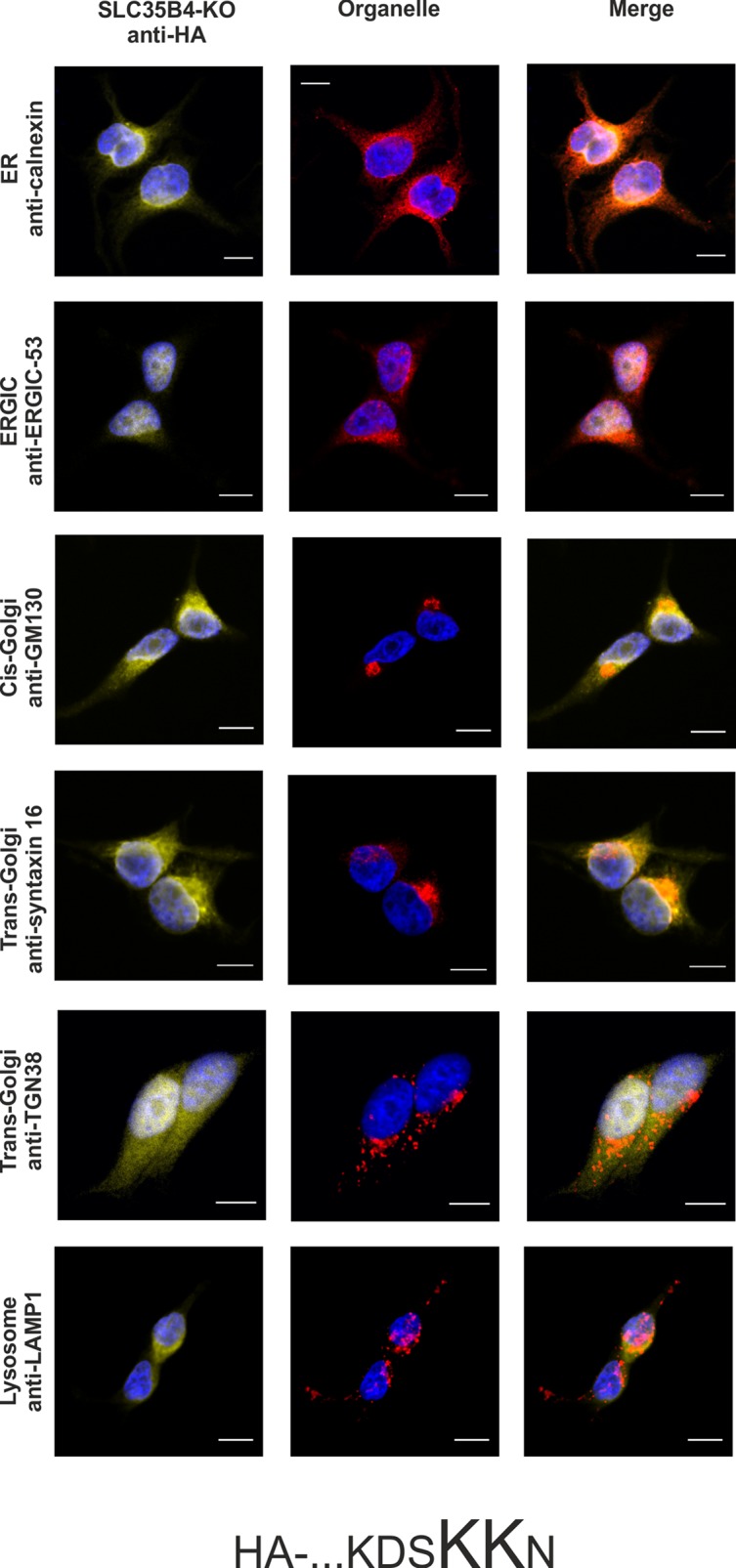
Subcellular localization of HA- …KDSKKN, human SLC35B4 with HA-epitope at the N-terminus in the knock-out HepG2 cells. Stably transfected cells, lacking functional *SLC35B4* gene (SLC35B4-KO), overexpressing SLC35B4 variants, were subjected to indirect immunofluorescent staining with anti-HA (green) and anti-organelle markers (Table 3) (red) antibodies. Cell nuclei were counterstained with DAPI. Scale bar 10 μm.

**Fig 8:**
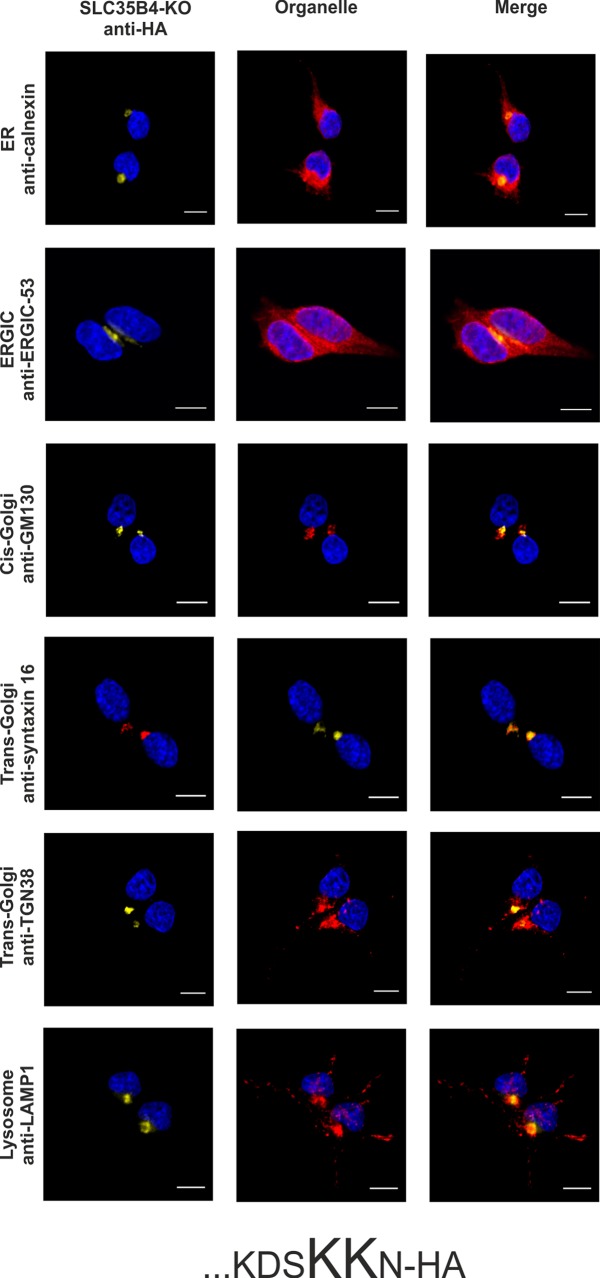
Subcellular localization of …KDSKKN-HA, human SLC35B4 with HA-epitope at the C-terminus in the knock-out HepG2 cells. Stably transfected cells, lacking functional *SLC35B4* gene (SLC35B4-KO), overexpressing SLC35B4 variants, were subjected to indirect immunofluorescent staining with anti-HA (green) and anti-organelle markers (Table 3) (red) antibodies. Cell nuclei were counterstained with DAPI. Scale bar 10 μm.

**Fig 9:**
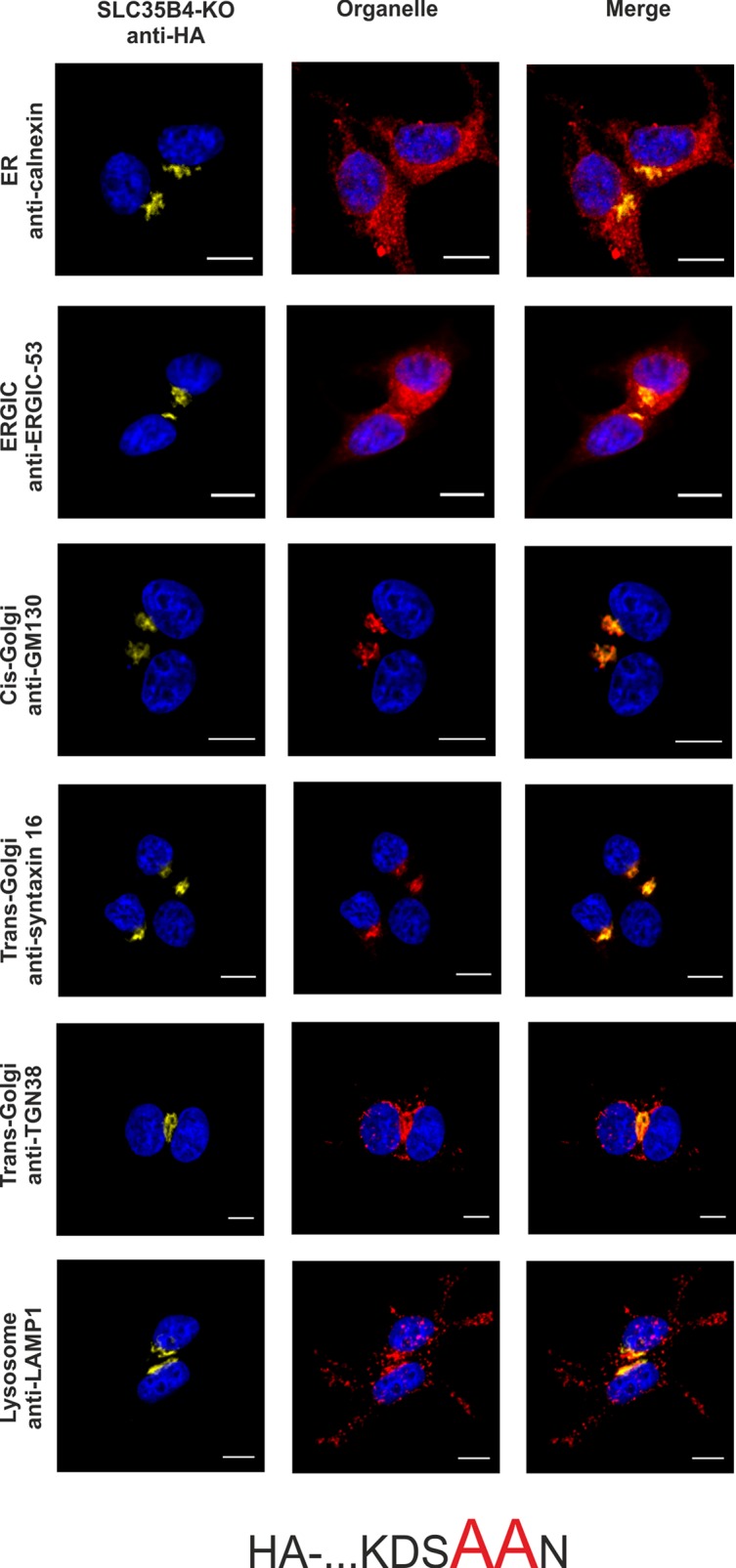
Subcellular localization of HA- …KDSAAN, human SLC35B4 K329AK330A with HA-epitope at the N-terminus in the knock-out HepG2 cells. Stably transfected cells, lacking functional *SLC35B4* gene (SLC35B4-KO), overexpressing SLC35B4 variants, were subjected to indirect immunofluorescent staining with anti-HA (green) and anti-organelle markers (Table 3) (red) antibodies. Cell nuclei were counterstained with DAPI. Scale bar 10 μm.

**Fig 10:**
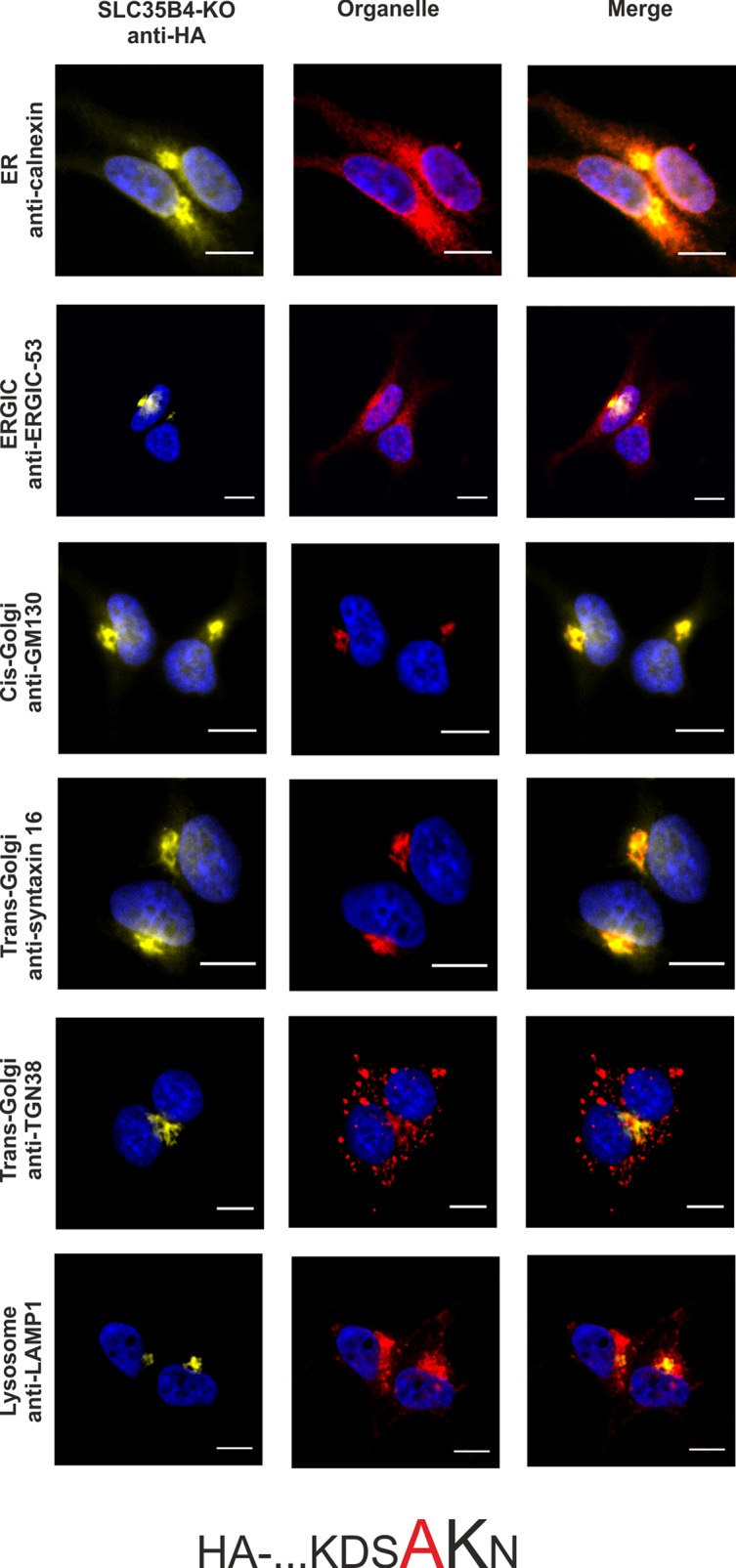
Subcellular localization of HA- …KDSAKN, human SLC35B4 K329A with HA-epitope at the N-terminus in the knock-out HepG2 cells. Stably transfected cells, lacking functional *SLC35B4* gene (SLC35B4-KO), overexpressing SLC35B4 variants, were subjected to indirect immunofluorescent staining with anti-HA (green) and anti-organelle markers (Table 3) (red) antibodies. Cell nuclei were counterstained with DAPI. Scale bar 10 μm.

**Fig 11:**
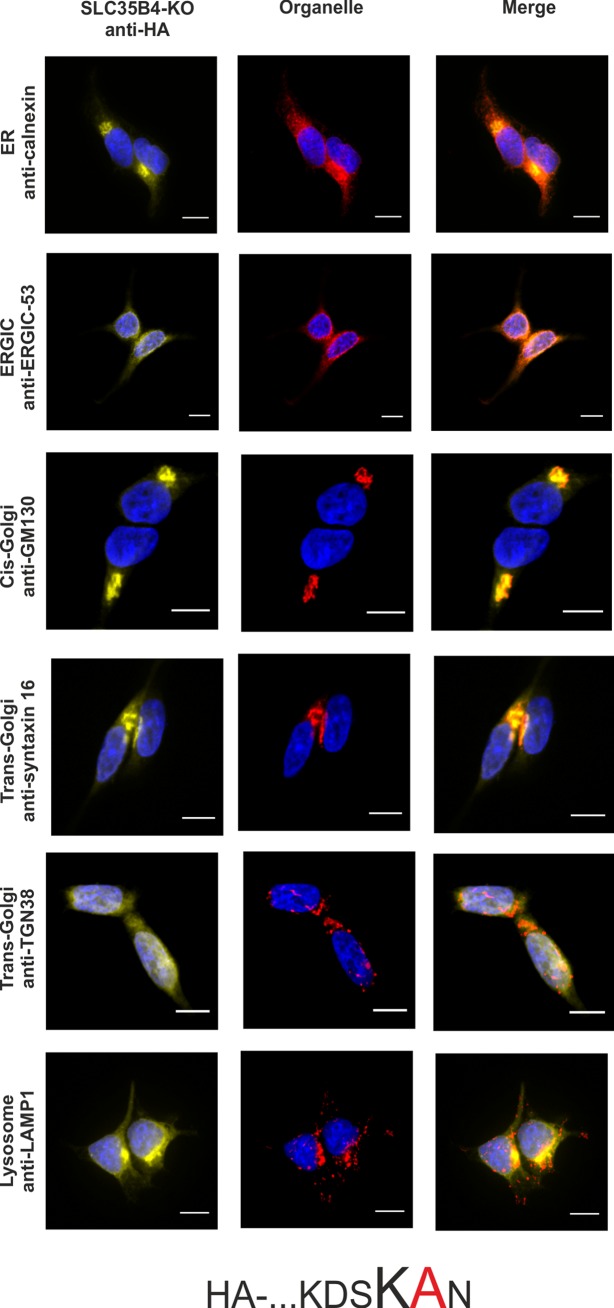
Subcellular localization of HA-…KDSKAN, human SLC35B4 K330A with HA-epitope at the N-terminus in the knock-out HepG2 cells. Stably transfected cells, lacking functional *SLC35B4* gene (SLC35B4-KO), overexpressing SLC35B4 variants, were subjected to indirect immunofluorescent staining with anti-HA (green) and anti-organelle markers (Table 3) (red) antibodies. Cell nuclei were counterstained with DAPI. Scale bar 10 μm.

**Fig 12:**
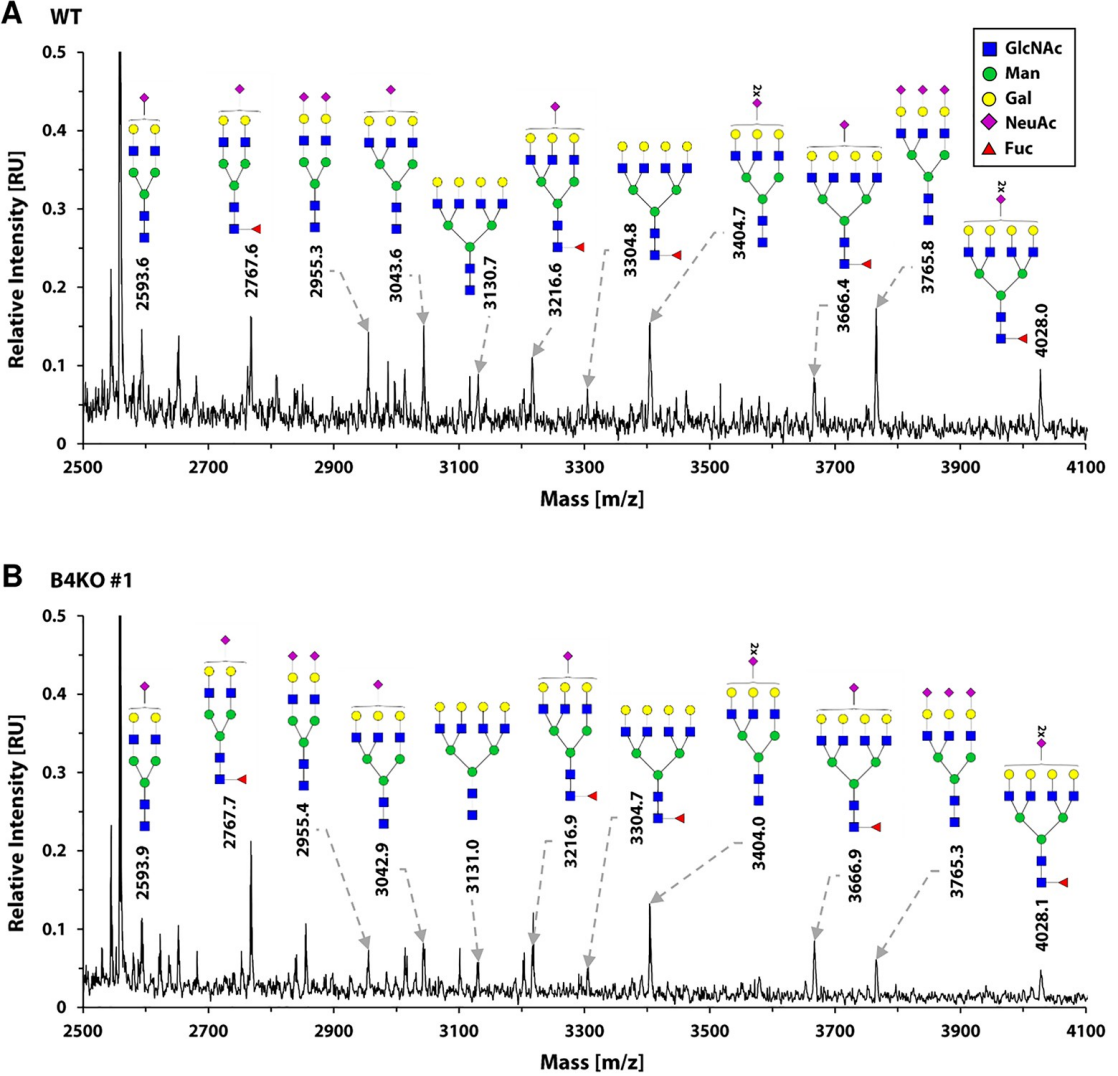
Analysis of *N*-glycans structures in the wild-type and SLC35B4 knock-out HepG2 cells. MALDI-TOF-MS characterization of permethylated 2-AB-*N*-glycans purified from wild-type (WT) (A) and SLC35B4-deficient (B4KO) (B) HepG2 cells. Spectra were offset and scaled. For clarity of presentation mass region was restricted to 2500–4100 Da to contain complex-type *N*-glycans only. Identified peaks were labeled with mass information and cartoon representations of putative *N*-glycan chemical structures (based on biosynthetic knowledge). Identification of phosphorylated or sulfated glycans was not attempted. All variations of differently branched and sialylated complex-type *N*-glycans detected in the wild-type cells were also detected in SLC35B4 knock-out clones showing that *N*-glycosylation profile was not affected. Representative data from two independent experiments are shown.

In order to avoid the eventual interference between the endogenous SLC35B4 and its recombinant variants we entirely reproduced the localization study in the knock-out cells. As shown in Fig 7–12, localization of HA-tagged SLC35B4 variants overexpressed in knock-out cells was similar as compared to the wild-type cells (Fig 1–5). Characterization of glycosylation of the mutated cells did not show significant changes as compared to the wild-type cells. This conclusion was drawn based on detailed MALDI-TOF-MS analysis of *N*- and *O*-glycans structures (Figs 4 and 5) and analysis of changes in levels of produced keratan sulfate and chondroitin-4-sulfate proteoglycans using immunoblotting (Fig 6).

The lack of significant changes in *N*-glycosylation was demonstrated based on qualitative mass spectrometry analysis (Fig 12). Using this method, in the sample prepared from the wild-type HepG2 cells we were able to detect eleven different species of mature oligosaccharides, including sialylated tetra-antennary structures (Fig 12A). Analogously, the same set of oligosaccharides was detected in the sample prepared from the SLC35B4 knock-out cells. This similarity suggests that, at least qualitatively, there are no significant differences in *N*-glycosylation pattern in the wild-type and SLC35B4 knock-out HepG2 cells. If SLC35B4 deficiency resulted in diminished UDP-GlcNAc import across organelle membranes one should expect changes in the presence of particular *N*-glycans.

**Fig 13:**
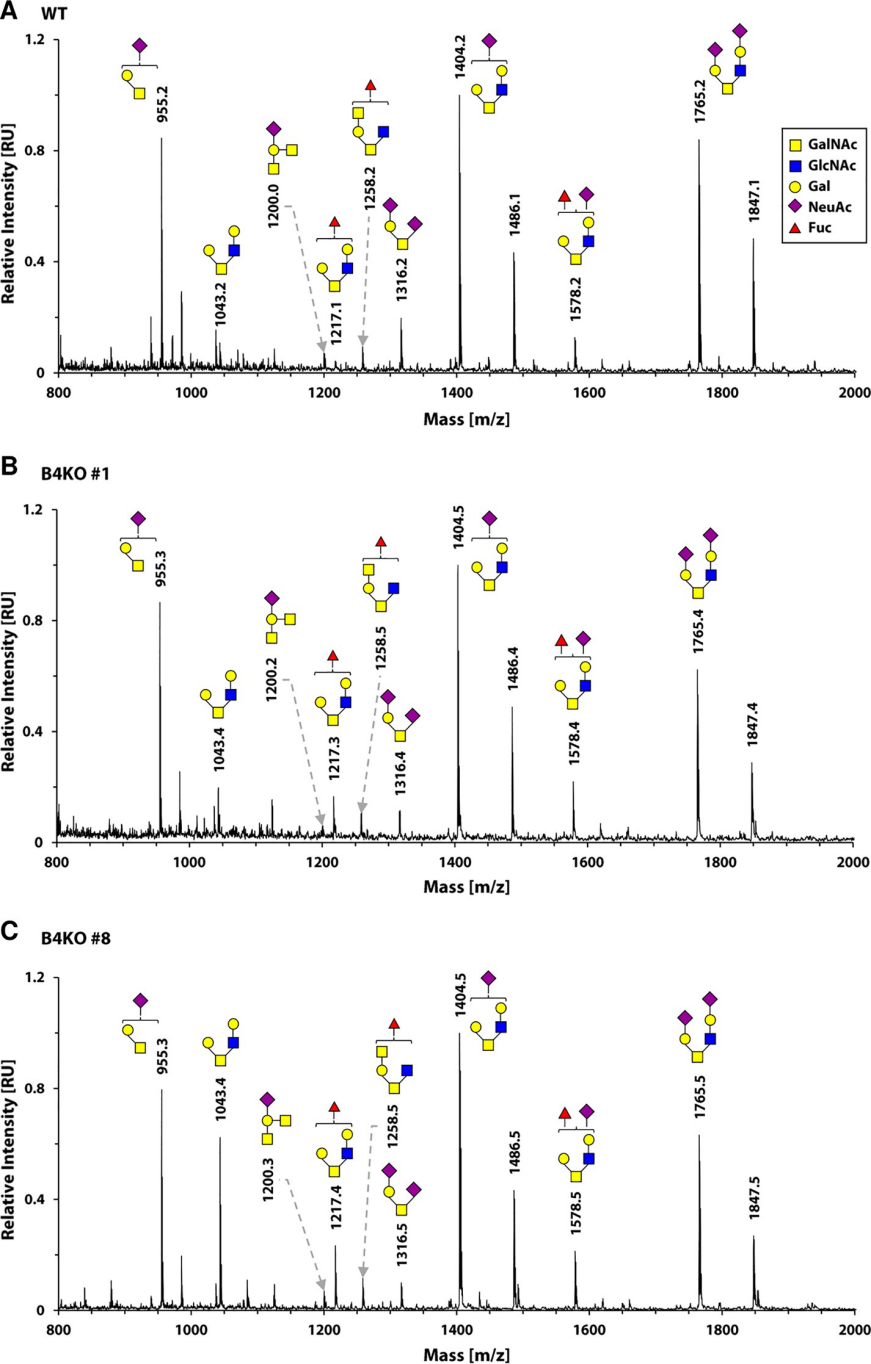
Analysis of *O*-glycans structures in the wild-type and SLC35B4 knock-out HepG2 cells. MALDI-TOF-MS characterization of permethylated Bn-*O*-glycans secreted to culture medium by wild-type (WT) (A) and SLC35B4-deficient (B4KO) (B,C) HepG2 cells. Spectra were scaled for maximum value. Identified peaks were labeled with mass information and cartoon representations of putative *O*-glycan chemical structures (based on biosynthetic knowledge). Identification of phosphorylated or sulfated glycans was not attempted. All variations of core 1- and core 2-type *O*-glycans detected in the wild-type cells were also detected in SLC35B4 knock-out clones showing that *O*-glycosylation profile was not affected. Representative data from two independent experiments are shown.

Similarly to *N*-glycosylation, we have also analyzed *O*-glycosylation pattern in both the wild-type and SLC35B4 knock-out HepG2 cells (Fig 13). Employment of a novel method of *O*-glycans analysis [24] in HepG2 cells allowed us to detect nine different oligosaccharide structures out of which seven were core-2 type and contained GlcNAc in their structures. The sets of oligosaccharides in both the wild-type (Fig 13A) and SLC35B4 knock-out (Figs 13B and 5C) HepG2 cells overlapped, suggesting that also *O*-glycosylation pathways were not affected by SLC35B4 deficiency.

As SLC35B4 is proposed also to be able to transport UDP-Xyl, its deficiency is expected to affect proteoglycans. In such a case, one should observe complete lack or at least a significant decrease in production of chondroitin-4-sulfate proteoglycans, since Xyl is essential for the formation of the tetrasaccharide linker by which glycosaminoglycan chains are attached to the protein core. In addition, the lack or decreased availability of UDP-GlcNAc in glycosylation-competent compartments of knock-out cells should result in truncated keratan sulfate proteoglycans or their depletion, similar to our previously published data on SLC35A3 knock-down MDCK cells [34].

**Fig 14:**
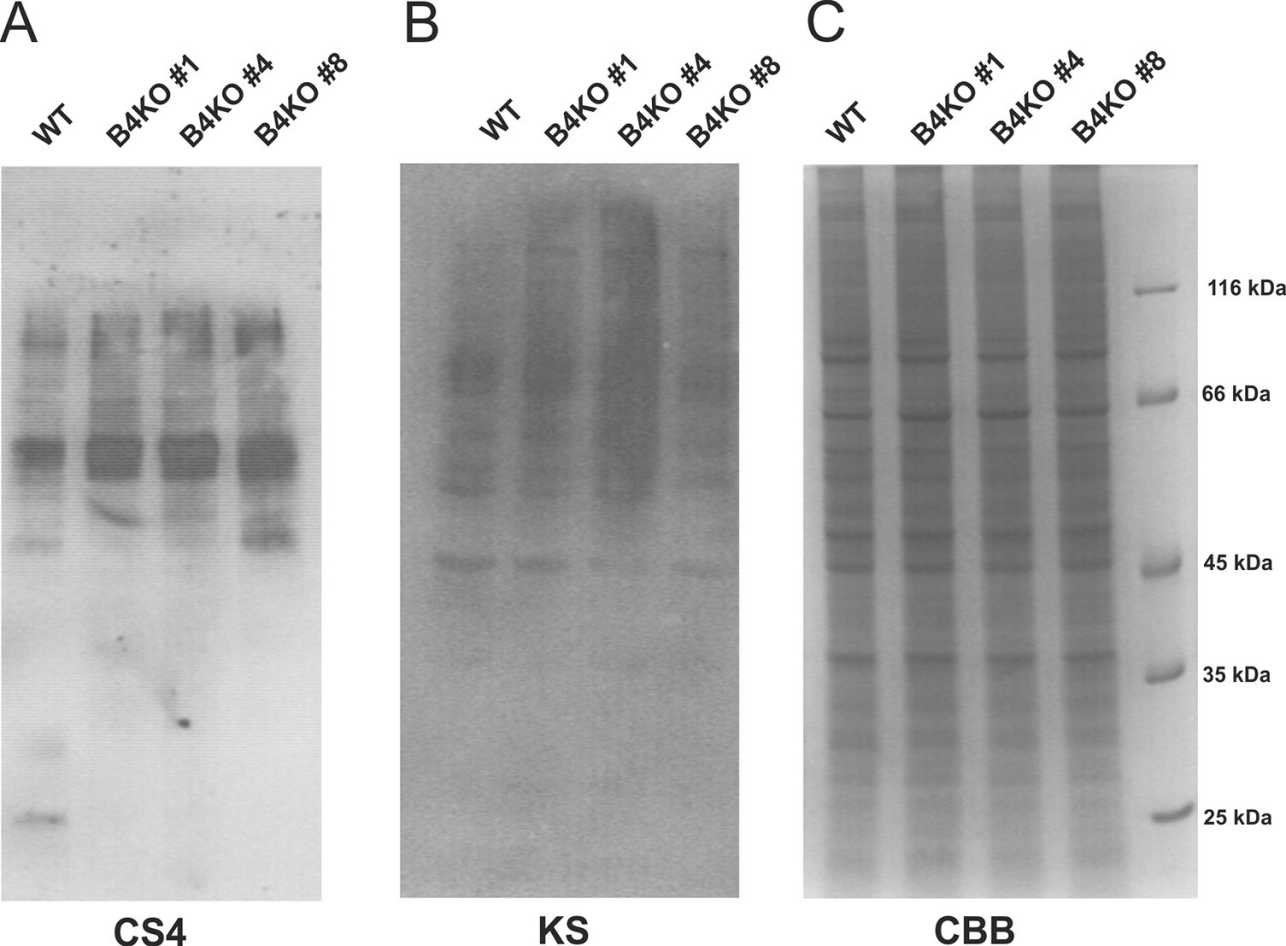
Analysis of proteoglycans. Proteoglycans present in cell lysates of wild-type (WT) and SLC35B4-deficient (B4KO) cells were subjected to SDS-PAGE, transferred onto nitrocellulose membrane and probed with anti-chondroitin-4-sulfate (C4S) (A) or keratan sulfate (KS) (B) antibodies. Protein loading was assessed using Coomassie Brilliant Blue G-250 (CBB) staining (C).

Therefore, to test the production of proteoglycans we performed Western blotting experiments on the wild-type and on the SLC35B4 knock-out HepG2 cell lysates using antibodies specific for chondroitin-4-sulfate or keratan sulfate (Fig 14). The amounts of samples loaded were normalized for total protein which was monitored by CBB staining. The immunostaining intensities of the samples prepared from the SLC35B4 knock-out HepG2 clones were even somewhat higher than the intensity of the sample derived from the wild-type cells which suggests that the proteoglycan content was not compromised by SLC35B4 deficiency.

In conclusion, our results show that overexpressed SLC35B4 is ER-resident. Using a systematic approach we demonstrated that placing a tag at the C-terminus compromises the protein’s ER-localization signal and causes its re-localization to the Golgi apparatus. More detailed analysis showed that analogous effect can be triggered by mutation of lysine at position 329 to alanine, suggesting that this amino acid is crucial for SLC35B4 correct localization. These findings provide evidence that one should use protein tagging with utmost caution, especially when conclusions on the biological function of examined proteins are drawn based solely on their localization. Our data also show that the knock-out of the *SLC35B4* gene does not influence the most essential and well-known glycosylation pathways utilizing UDP-Xyl and UDP-GlcNAc as substrates. Based on our findings we postulate a different, as yet uncovered role of SLC35B4 in human metabolism.

## Supporting information

S1 Fig Schematic representation of *SLC35B4* gene inactivation carried out through CRISPR-Cas approach and the gene knock-out confirmation by genomic DNA sequencing. Exon #1 of *SLC35B4* gene is marked in red, ATG start codon is marked in yellow (wild-type only; wt), deletions of 270-bp in three knock-out (KO) clones are shown as dashed lines (—), sequences used to design primers and amplify the genomic fragment are marked in gray, sequences used to design guide RNAs for CRISPR-Cas knock-out plasmids are shown in bold and underlined.
(DOCX)
