# Supplementary material for: Lysine at position 329 within a C-terminal dilysine motif is crucial for the ER localization of human SLC35B4
Source: PLoS One. 2018 Nov 20;13(11):e0207521. doi: 10.1371/journal.pone.0207521 (PMC6245738; doi:10.1371/journal.pone.0207521)
Supplement: S1 Fig — Exon #1 of SLC35B4 gene is marked in red, ATG start codon is marked in yellow (wild-type only; wt), deletions of 270-bp in three knock-out (KO) clones are shown as dashed lines (—), sequences used to design primers and amplify the genomic fragment are marked in gray, sequences used to design guide RNAs for CRISPR-Cas knock-out plasmids are shown in bold and underlined. (DOCX) [file pone.0207521.s001.docx]

*1* 5’- UTR  *120*

HepG2 wt GTCCTCTCTGGCGGAGCTGCCTGGCGGAAGCGGGAACGTCGCATCCTGAGGTAAAGGTGCACGGCATCCTGGGACATGTAGTCTGGCCGGGGCTCGGACGCCCCCTCGGATGAATGGGAC

B4_KO_1 GTCCTCTCTGGCGGAGCTGCCTGGCGGAAGCGGGAACGTCGCATCCTGAGGTAAAGGTGCACGGCATCCTGGGACATGTAGTCTGGCCGGGGCTCGGACGCCCCCTCGGATGAATGGGAC

B4_KO_4 GTCCTCTCTGGCGGAGCTGCCTGGCGGAAGCGGGAACGTCGCATCCTGAGGTAAAGGTGCACGGCATCCTGGGACATGTAGTCTGGCCGGGGCTCGGACGCCCCCTCGGATGAATGGGAC

B4_KO_8 GTCCTCTCTGGCGGAGCTGCCTGGCGGAAGCGGGAACGTCGCATCCTGAGGTAAAGGTGCACGGCATCCTGGGACATGTAGTCTGGCCGGGGCTCGGACGCCCCCTCGGATGAATGGGAC

*121 240*

HepG2 wt CGAAGCTGACTGCGAACTACAGCTTCTTGGCAGCGTCGGTGTTGGCCGCGGGAGAAGGGGAGACCGCGGCGGCCCCCAGTGAGAGCGGCTTTCCAGGACGGTGCGATGTGCTGCGCAGCG

B4_KO_1 CGAAG-------------------------------------------------------------------------------------------------------------------

B4_KO_4 CGAAG-------------------------------------------------------------------------------------------------------------------

B4_KO_8 CGAAG-------------------------------------------------------------------------------------------------------------------

*241* **[** **Exon 1***……………………………………..360*

HepG2 wt AAGAGGCAGGAGGCCGGCTTCCTGGGGTAGCGGTACAGGCGGGCGCTTACTCTGTGCGCTTGCTTCCCCAACCCTGCACCGGCC***ATG****CGCCCGGCCTTGGCGGTGGGCCT****GGTGTTCGCA***

B4_KO_1 ------------------------------------------------------------------------------------------------------------------------

B4_KO_4 ------------------------------------------------------------------------------------------------------------------------

B4_KO_8 ------------------------------------------------------------------------------------------------------------------------

*361*………………………**Exon 1**……………………………………………………………**]** Intron 1 *480*

HepG2 wt ***GGCTGCTGCA****GTAA****CGTGATCTTCCTAGAGCTCC****TGGCCCG*GTGAGTGACCCGCTCGGGCCGCACCCGGTCTCCCGGGGCGGGGCGGAGGAAGCCAGAGGAGAGGTCCCCCCGGGACGCG

B4_KO_1 -----------------------------------*GGCCCG*GTGAGTGACCCGCTCGGGCCGCACCCGGTCTCCCGGGGCGGGGCGGAGGAAGCCAGAGGAGAGGTCCCCCCGGGACGCG

B4_KO_4 -----------------------------------*GGCCCG*GTGAGTGACCCGCTCGGGCCGCACCCGGTCTCCCGGGGCGGGGCGGAGGAAGCCAGAGGAGAGGTCCCCCCGGGACGCG

B4_KO_8 -----------------------------------*GGCCCG*GTGAGTGACCCGCTCGGGCCGCACCCGGTCTCCCGGGGCGGGGCGGAGGAAGCCAGAGGAGAGGTCCCCCCGGGACGCG

*481* Intron 1 *600*

wt CCCACGCCCTGTCCCCACCCCGCCGCCCACGGAGCCCGGGGCAGGACGGCCTGAGCCCCCCAGCAATCCGTCCTGTCTGGGCAGCCTGGGACCCCGCTGAGTCCGCTGCTTCCCAAAACT

B4_KO_1 CCCACGCCCTGTCCCCACCCCGCCGCCCACGGAGCCCGGGGCAGGACGGCCTGAGCCCCCCAGCAATCCGTCCTGTCTGGGCAGCCTGGGACCCCGCTGAGTCCGCTGCTTCCCAAAACT

B4_KO_4 CCCACGCCCTGTCCCCACCCCGCCGCCCACGGAGCCCGGGGCAGGACGGCCTGAGCCCCCCAGCAATCCGTCCTGTCTGGGCAGCCTGGGACCCCGCTGAGTCCGCTGCTTCCCAAAACT

B4_KO_8 CCCACGCCCTGTCCCCACCCCGCCGCCCACGGAGCCCGGGGCAGGACGGCCTGAGCCCCCCAGCAATCCGTCCTGTCTGGGCAGCCTGGGACCCCGCTGAGTCCGCTGCTTCCCAAAACT

*601* Intron 1 *720*

wt GCGCACCGGCGGCAGTCCCGCCTAACAGTTTCAAAGCATTCCTGCGAGGGGAAGACTCAGGGTGTTGCTGTCGTCACCCCTTTTCCACACTCGGGGGGTATTGAAGTCTGAAGAGTTTTT

B4_KO_1 GCGCACCGGCGGCAGTCCCGCCTAACAGTTTCAAAGCATTCCTGCGAGGGGAAGACTCAGGGTGTTGCTGTCGTCACCCCTTTTCCACACTCGGGGGGTATTGAAGTCTGAAGAGTTTTT

B4_KO_4 GCGCACCGGCGGCAGTCCCGCCTAACAGTTTCAAAGCATTCCTGCGAGGGGAAGACTCAGGGTGTTGCTGTCGTCACCCCTTTTCCACACTCGGGGGGTATTGAAGTCTGAAGAGTTTTT

B4_KO_8 GCGCACCGGCGGCAGTCCCGCCTAACAGTTTCAAAGCATTCCTGCGAGGGGAAGACTCAGGGTGTTGCTGTCGTCACCCCTTTTCCACACTCGGGGGGTATTGAAGTCTGAAGAGTTTTT

*721* Intron 1 *778*

wt CACATGACCAGTCCAAGGTCGGCAGAGAGCTAGTGTCAGAACCAAGGTTCCTTCACCA

B4_KO_1 CACATGACCAGTCCAAGGTCGGCAGAGAGCTAGTGTCAGAACCAAGGTTCCTTCACCA

B4_KO_4 CACATGACCAGTCCAAGGTCGGCAGAGAGCTAGTGTCAGAACCAAGGTTCCTTCACCA

B4_KO_8 CACATGACCAGTCCAAGGTCGGCAGAGAGCTAGTGTCAGAACCAAGGTTCCTTCACCA
